# Understanding HPV-positive women’s needs and experiences in relation to patient-provider communication issues: a qualitative study

**DOI:** 10.1186/s12913-021-06283-w

**Published:** 2021-03-31

**Authors:** Kowsar Qaderi, Mehrnaz Geranmayeh, Farnaz Farnam, Shahrzad Sheikh Hasani, Seyedeh Tahereh Mirmolaei

**Affiliations:** 1 Reproductive Health Department, School of Nursing and Midwifery, Tehran University of Medical Sciences, Eastern Nosrat st. Tohid sq, Tehran, 141973317 Iran; 2 Gynecology Oncology Department, Imam Khomeini Hospital Complex, Tehran University of Medical Sciences (TUMS), Tehran, Iran

**Keywords:** Papillomavirus infections, Health care provider, Expectations, HPV, Women, Cervical Cancer screening, Needs, Qualitative, Education

## Abstract

**Background:**

HPV testing has been integrated in cervical cancer screening program. Patient-providers relationship is extremely important to improve cervical cancer screening outcomes. This qualitative study aims to understand HPV-positive women’s needs and preferences about HCPs and patient-provider communication based on their experiences of accessing primary and specialized care.

**Methods:**

We conducted 40 semi-structured interviews with HPV-positive women. Recorded interviews transcribed and analyzed using conventional content analysis approach.

**Results:**

The analysis of the data led to the extraction of three main categories, including: provider’s communication and counseling skills, commitment to professional principles, and knowledgeable and competent provider. Women needed understandable discussion about HPV, emotional support and acceptance, receiving HPV-related guidance and advice, and some considerations during clinical appointments. Women needed HCPs to treat them respectfully, gently and with non-judgmental attitude. “Precancerous” and “high-risk” words and watching colposcopy monitor during procedure had made women anxious. Weak referral system and limited interactions among gynecologists and other HCPs highlighted by participants.

**Conclusion:**

The results of this study, based on the experiences and perceptions of HPV women receiving health care, contain messages and practical tips to healthcare providers at the primary and specialized levels of care to facilitate patient-provider communication around HPV. Providers need to approach the discussion of HPV with sensitivity and take individual needs and preferences into account to improve the HPV-positive women’s healthcare experience.

**Supplementary Information:**

The online version contains supplementary material available at 10.1186/s12913-021-06283-w.

## Introduction

Human Papillomavirus is a common sexually transmitted infection, with potentially serious health consequences like cervical cancer (CC) [1] which is preventable through the availability of HPV vaccination and the possibility of high-risk HPV screening test [2]. Health organizations place a higher priority on HPV testing in developing countries where HPV vaccination coverage is poor [2].

In Iran, the national HPV vaccination program has not been started but Gardasil can be purchased around $100 in some pharmacies. The national CC screening program recommends co-testing (Pap smear and HPV testing) for women aged 30 to 59 every 5 years [3]. Although co-testing is performed in all primary healthcare centers, public/private insurance plans do not cover it. There are limited government-funded centers in provincial capitals that charge less for CC screening and diagnostic tests.

Research indicates that participating in HPV testing and receiving an HPV diagnosis can create adverse psychosocial responses [4–8]. To avoid anxiety and psychological distress, with more women being screened for or diagnosed with HPV, patient education and counseling efforts are increasingly important and more attention should be given to provider-patient communications [9] and information provision for HPV-infected women [10].

It has been sufficiently documented in literature that successful communication leads to greater adherence [11, 12] and adherence to recommended cervical screening guidelines is associated with better clinical outcomes [13]. Good patient-physician communication and patient-centered care practices can improve health literacy and patient-physician satisfaction [14]. Also, in primary care, trust and the patients’ optimistic believes about healthcare providers (HCPs) lead to greater patient adherence to care and better disclosure of sensitive information [12, 15]. Emotional and informational support and a private counseling environment also increase patient satisfaction [16, 17]. Despite the obvious benefits of patient-centered care, lack of training in communication skills and time pressure are major challenges for providers [18].

Previous studies have investigated HPV communication needs and gaps between HCPs and vaccine-eligible adolescent or HPV-related cancer patients [19–25]. Head and neck cancer (HNC) patients requires appropriate communication about HPV and high-quality information from healthcare providers [26].

A research has shown informational challenges and communication gaps occurred for women diagnosed with CIN in the moments of the CC screening in primary care, waiting time until referral to specialized care, first consultation in specialized care, and after consultation in specialized care [27]. Relatively little research has also shown that healthcare providers have knowledge gaps [28] and there is no adequate flow of HPV-related information between HCPs and women [29].

Counseling HPV patients presents particular challenges for both physician and patient [30] particularly in societies where HPV is infrequent and attached with intense stigma. It would be valuable to provide a deep understanding of HPV-positive women’s challenges, perspectives and experiences of communicating with healthcare providers in primary and specialized healthcare levels. Qualitative inquiries uncover trends in thoughts and reveal underlying views, opinions, preferences and expectations [26]. Therefore, we designed a qualitative study to understand HPV-positive women’s needs, perspectives and preferences about HCPs and patient-provider communication based on their experiences and perceptions of accessing primary and specialized care.

## Methods

Qualitative face-to-face semi-structured interviews were conducted with 40 Iranian women received positive high-risk HPV result and content analysis approach was used to data analysis. Th interviews were conducted from September 2018 to December 2019 at Valiasr outpatient referral gynecology-oncology clinic in Tehran which serves a large population of women across the country. So, the time given to each patient is limited. The services in this clinic (including colposcopy) are very low-priced and offered to all patients, regardless of their insurance coverage.

The clinic is directed by five oncologist-gynecologists and two midwives. The clinic coordinator (a midwife) sent all HPV-positive women (either only hrHPV or both high-risk and low-risk strains) to the interviewer (KQ-female- no contact with participants) in a quiet, comfortable room to provide information on the study’s goals and methods and invite qualified women to participate in the study. Women were eligible for interview if they were over 18 years of age with a heterosexual partnership; had no serious illness (including CC) and were willing to share experience. A purposeful maximum variation sampling was used to recruit information-rich Persian-speaking HPV-positive candidates (*N* = 40) with a diverse age, marital status, education, ethnic, cultural and religious backgrounds, and socio-economic status (Table 1).

**Table 1:** HPV genotypes, cytology results and demographic characteristics of participants

Characteristics	(*n* = 40)
**HPV Genotypes**	
High Risk	21(52.5%)
Mixed (Low & High Risk)	19(47.5%)
**Pap Test Results**	
Normal	13(32.5%)
ASCUS^a^	12(30%)
LSIL^b^	11(27.5%)
HSIL^c^	4(10%)
**Age**	
< 30 years	6(15%)
30–39 years	26(65%)
40–49 years	7(17.5%)
≥ 50 years	1(2.5%)
**Marital status**	
Married	17(42.5%)
Unmarried	23(57.5%)
**Education level**	
lower Intermediary	16(40%)
University- Bachelor	11(27.5%)
Master or PhD	13(32.5%)
**Occupation**	
Housewife	15(37.5%)
Employed	25(62.5%)

^a^Atypical Squamous Cells of Undetermined Significance

^b^Low-Grade Squamous Intraepithelial Lesion

^c^High-Grade Squamous Intraepithelial Lesion

Interviews started with demographic context and screening history (Additional file 1). We sought to understand HPV-positive women’s perspectives and preferences about provider/patient communication based on their experiences. Two women declined to participate, choosing not to discuss HPV. Three pilot interviews (included in study) were conducted to improve questions. Memos helped create next interview questions. In-depth interviews with consent of participants were recorded (35–90 min), transcribed verbatim, and collected until data saturation was achieved.

Midwives and gynecologists take cervical examinations in Iran. We also interviewed ten providers (Table 2) at primary and specialized care levels (15–20 min) if they had more than 5 years of experience providing services to HPV-infected women and were willing to participate in the interviews to confirm HPV-positive women’s needs and preferences about provider/patient communication.

**Table 2:** Demographic characteristics of interviewed providers

ID Number	Healthcare Provider	Age	Sex	Workplace	Marital Status	Work experience (Years)	Number of children
1	Oncologist-gynecologist	43	Female	Valiasr Clinic	Married	8	2
2	Oncologist-gynecologist	45	Female	Private office	Married	12	1
3	Gynecologist	42	Female	Valiasr Clinic	Married	12	2
4	Gynecologist	46	Female	Private Clinic	Married	18	1
5	Gynecologist	38	Female	Valiasr Clinic	Married	11	1
6	Midwife (Bachelor)	35	Female	Private Clinic	Single	10	0
7	Midwife (Master)	43	Female	Valiasr Clinic	Married	14	1
8	Midwife (Bachelor)	31	Female	Valiasr Clinic	Single	7	0
9	Midwife (Master)	47	Female	Private office	Married	15	2
10	Virologist (Lab Director)	48	Male	Private Laboratory	Married	16	2

Conventional qualitative content analysis method, described by Burnard et al. [31], was performed using the trial version of MAXQDA-10 data-management software simultaneously with data collection. Initially, interview transcripts, memos, and field notes were integrated, and two coders (KQ and STM) read transcripts repeatedly to formulate a general understanding of entire data. Open coding was conducted. The extracted codes were combined with similarities and variations. In higher abstraction levels, sub-categories with related content were interpreted into the main categories.

The accuracy of this qualitative study was assured based on the four parameters of Guba and Lincoln: credibility, reliability, confirmability, and transferability [32, 33]. The credibility criterion was achieved by prolonged engagement and member checking. Peer debriefing and external checking ensured performance confirmability and reliability. Two observers reviewed all transcripts, codes, and categories. Lastly, this process concluded with several discussions among the research team on areas of disagreement before achieving final consensus. Considering maximum variation during sampling, we tried to enhance transferability. In qualitative study, generalizability was labeled as a full description of setting, participants, and categories in rich detail through the outside reader’s lens. To achieve reliability, the study procedure was described comprehensive. Direct quotes from participants have presented.

This study is coming from a Ph.D. thesis on Reproductive Health, a mixed method study that was reviewed and accepted by the Ethics Committee of Tehran University of Medical Sciences (date: 29 Oct 2018; registration number: IR.TUMS.FNM.REC.1397.139). Valiasr hospital managers willingly facilitated the study. Written informed consent obtained from all participants.

## Results

Participant characteristics shown in Table 1 demonstrate the heterogeneity of sample. Women averaged 34.07 years (21–52 years). All had visited healthcare providers regarding their HPV infection in the 12 months preceding the interview. Half received a diagnosis from a gynecologist, 35 % from a primary healthcare provider (midwife), and remain from a general practitioner. All saw a provider other than the one who initially diagnosed them.

Women were asked about their experiences, needs and preferences about their HCPs and patient-provider communication with through a series of questions (Additional file 1). The analysis of the data led to the extraction of three main categories, including: provider’s communication and counseling skills, commitment to professional principles, and knowledgeable and competent provider (Table 3). Details in parentheses following quotes represent the participant’s identification number (W. = HPV-positive Woman, Pr. = Provider).

**Table 3:** Needs and Perceptions of Iranian HPV-positive women about receiving health care

Categories	sub-categories	Example of codes
**1. Communication and Counseling Skills**	a. Understandable discussion about HPV	Skills in breaking bad news Providing adequate HPV-information with understandable, colloquial language Taking time to answer the patient’s questions Delivering HPV-information gradually Avoid exaggerating or underestimating HPV risks Listen intently to the patient and not to dominate the conversation
	b. Emotional support and acceptance	Paying attention to the patients’ feelings and concerns Encouraging words to strengthen the patient’s spirit The need for compassionate doctors in the medical centers Doctor’s positive and hopeful attitude towards HPV clearance
	c. Need to receive HPV-related guidance and advice	Explaining the risk and providing advice and solutions to strengthen immunity and reduce the cancer risk Discussing sexual practice, diet, alcohol and tobacco prohibitions or modification Vaccine recommendations
	d. Clinical appointment considerations	Not requesting for HIV and hepatitis tests at the first visit Not sharing the colposcopy monitor with patient unless she wants Explaining colposcopy before performing it Not asking/ reporting low-risk HPV strains
**2.Commitment to Professional Principles**	a. Confidentiality and privacy	Visiting patients one by one Clinic’s staff awareness of the patient’s secrecy patient privacy in the gynecology clinics Consider cultural sensitivity
	b. Ethics in research and practice	Avoid try and error in patients’ management Obtaining written informed consent from patients in cases of doing research
	c. Non- judgmental language and behaviors	Adopt non-judgmental attitude toward patient’s sexual behavior Building mutual trust/ Being honest with patient
	d. Considering the patient’s financial issues	Avoiding humiliating behaviors towards poor patients Introducing patients to governmental-funded services instead of private centers Adopt scientific approaches Avoid prescribing self-made medications
**3. knowledgeable and competent provider**	a. Adherence to screening guidelines	Adopt scientific management and avoid overuse tests Screening eligible woman Adherence to test intervals Follow-up according to the national cervical cancer guideline
	b. Addressing uncertainties and misconceptions	Discussing the current gaps in HPV-knowledge Citing conflicting views Communicating intentionally inexact about infection source Avoid exaggerating about HPV transmission by overusing protective equipment Avoid Instilling fallacy that HPV has a treatment (self-made suppositories, fungi, and probiotic products) HCPs’ participating in retaining programs
	c. Taking multidisciplinary approach	HPV women’ need for multidisciplinary team Wandering from the duality of therapists’ opinions Unnecessary patients’ referrals (to be on the safe side) Women’s wandering to find required specialist (Gynecologist-Infectious disease specialist-Oncologist-Urologist-Dermatologist-ENT specialist-Dentist) Counseling about anti-wart treatments (in oral, anal and genital area) Refer women with genital warts to dermatology clinics

### Provider’s communication and counseling skills

Women diagnosed with HPV implied communicating preferences and needs in four sub-categories: understandable discussion about HPV, emotional support and acceptance, need to receive HPV-related guidance and advice, and clinical appointment considerations.

#### Understandable discussion about HPV

Women needed face-to-face delivery of their results (HPV/cytology) to receive complex information about HPV, its types, symptoms, transmission, prevalence, consequences and treatments.

Several participants reported that the providers had broken the positive-results news in a way that has made them anxious and shocked.

*"When I was told 'You have a problem but it's not cancer', it frightened me to death. I was bursting crying ... I didn't understand what she [doctor] was talking about."*(W.16)

Women made a few points that they believed would make the discussion about HPV more understandable. Women needed HCPs to use plain language, avoid underestimating and over-simplifying the disease, stick to the factual information about HPV. The terminology commonly employed by physicians, were mostly unfamiliar to women. Few women indicated that when a doctor considers HPV insignificant, they feel ignored.

*"I was so scared. My doctor said: 'cancer patients don't mourn like you. HPV is not that important'. I think insomuch she sees cancer it’s gotten trivial for her."(*W.26)

To prevent misunderstanding and ensure patient awareness, participants also noted that gradually transmitting the information in multiple appointments helped them with coping and asking their questions.

Few women stated that comparing the incidence of cervical cancer and HPV infections prevalence (to clarify the cancer probability in HPV-infected women) has decreased their fear of cancer. Several women were terrified after hearing the term “precancerous/high-risk”. About the initial patient’s fear of cancer, a provider stated:

*"Pointing out that HPV is common and most women are unaware of their infection changes women's attitudes toward diagnosing HPV from a threat to an opportunity to prevent cancer."*(Pr.8)

Some patients expressed dissatisfaction with the time physicians spend to address their concerns. *“Very few doctors make time for the patient and talk to them patiently. Our services are not really good compared to some developed countries.”*(W.23).

Few women reported that their primary HCPs had referred them to the specialized clinic without explaining their health problems. Women’s questions on HPV were often inadequately answered, particularly in specialized governmental crowded referral clinics. *“No gynecologist has the patience and time to answer my questions.”*(W.12).

#### Emotional support and acceptance

Our respondents were more satisfied with compassionate providers and saw them as a source of emotional support.

*"Every patient needs a good doctor. The patient's thoughts and beliefs forms based on his/her doctor's words. My doctor treated me well and gave me hope and spirit."*(W.2)

According to participants’ point of view, the cold, annoying, and repulsive behaviors of the provider/doctor hinders successful follow-up and treatment. Women’s feelings and concerns needed to be accepted by their HCPs. women found the doctor’s excessive self-protection behaviors (e.g., wearing three pairs of gloves) repulsive, HCPs attributed them to the insufficient knowledge of their colleagues.

#### Need to hear advice and guidance

Women indicated that encountering this fact that “HPV has no cure”, they have felt powerless because they can’t do anything to maintain their health.

*"I preferred my doctors to give me a tough solution/treatment rather than leaving me alone with the fact that there was no cure for HPV. Some providers have simple, useful, and inexpensive tips to strengthen immunity of patients (exercise, alcohol and tobacco prohibitions and healthy diet).*"(W.2) In this regard a provider stated:

*"When I say most HPV infections will clear up or become undetectable on their own, some women ask me: 'Why hasn't my infection cleared?' They usually ask me if there is a test for checking the immune system or what they can do to reduce the risk of cancer to the negligible levels.*” (Pr.5)

Although women needed advice on sexual health, the participants indicated that they feared bringing up the subject. A gynecologist-oncologist mentioned: *“Due to cultural sensitivities, neither patients nor doctors are inclined to speak about sexual issues.”*(Pr.1).

Women with genital warts needed additional information on surgical/medical anti-wart treatments.

#### Clinical appointment considerations

The uncomfortable or painful gynecological examination was a problem that several women described as a factor that prevented them from returning to screening.

*"I no longer go to screening due to the intense pain I experienced."*(W.22)

Before performing the colposcopy procedure, women needed verbal or written information. Few women reported that watching the colposcopy monitor during the procedure made them terrified.

*"I can't get that frightening colposcopy picture off my mind!"*(W.9)

They also preferred HIV and hepatitis tests not be asked at the very first appointment.

### Commitment to professional principles

Some of the needs emerged by women with positive HPV results were related to observing professional principles. Their statements showed that some HPV-positive women had negative experiences and wanted better healthcare providers’ performances in these areas (confidentiality and privacy, research ethics observance, non-judgmental language and behaviors and considering the patient’s financial issues).

#### Confidentiality and privacy

Participants noted that in addition to physicians, any provider dealing with sexually transmitted patients must be aware of and committed to privacy and secrecy.

“*I think OB/GYN visits require the most privacy. I went to a famous GYN's clinic which besides me, there were other patients in the room. Imagine sitting next to a complete stranger who might hear and talking about sensitive topics that are very difficult to discuss... I was so embarrassed.”* (W.10)

Several women mentioned that some clinic’s secretaries have talked about their condition (HPV) in the presence of other patients.

#### Ethics in research and practice

Women stated several doctors had recommended or prescribed unproven medications for HPV cure e.g., probiotics and herbal vaginal suppositories. Patients were not sure if it had been a research project or not. A woman stated: *“I don’t know, maybe my doctor is doing try and error”* (W.17).

#### Non- judgmental language and behaviors

Participants were less satisfied with provider/physicians who highlight stigma by their judgmental behaviors and language. Women did not trust such HCPs and were reluctant to share their private information with them. Few women attributed the provider’s excessive self-protection behaviors to religious issues related to impurity.

#### Considering the patient’s financial issues

Participants indicated lack of insurance coverage for diagnostic and therapeutic services. Patient pessimistic believes about financial misconducts of HCPs revealed in some interviews. *“I paid 10,000,000 IRR for a colposcopy in a private office. If my doctor had referred me here [Valiasr], I could’ve had a colposcopy for 400,000 IRR! She deliberately did not refer me here!”*(W.14).

Some women were upset of HCPs who treat them based on their economic status. Few women with low socio-economic status felt being rejected by their HCPs.

*"My doctor recommended a colposcopy; After she had realized I had no money for it, she stopped explaining it and did not answer my questions anymore"*(W.17)

### Knowledgeable and competent provider

Knowledgeable and competent HCP is among the three main needs and preferences of women infected with HPV. Participants indicated to the importance of HCPs HPV-knowledge in three main sub-categories as follows:

#### Adherence to screening guidelines

Our findings showed that although some women were eligible for screening, they were not invited to be screened by a midwife/gynecologist at the primary or specialized level. A 34-years-old HPV-screening eligible woman reported:

*"After my husband's genital warts, I went to a gynecologist who said: 'It doesn't matter. Since you don't have a lesion, you don't need HPV testing."*(W.31)

A few participants, on the other hand, reported another form of guideline discordant that some gynecologists appeared to overuse screening tests (upon the patient’s request) to provide greater reassurance. A woman stated:

*"My doctor has recommended me to have GYN examinations once every two months."* (W.12)

#### Addressing uncertainties and misconceptions

Women noted that uncertainties such as HPV-vaccination of HPV-infected people should be addressed in HPV-discussion to prevent them from confusing and wandering.

*"We don't know whether we should take HPV-vaccine or not."* (W.6)

Participants indicated that physicians’ conflicting opinions on HPV vaccination and using condoms in those already infected with HPV were among the reasons to their frequent visits. Regarding HPV transmission concerns and questions of women, a gynecologist stated:

*"I explain (my patients) intentionally vague about HPV transmission; I try to convince them that they could have contracted it in almost any way."*(Pr.2)

Several women reported their HCPs have used extra-protective equipment such as wearing three pairs of surgical gloves indicating a misconception that non-sexual transmission of the virus is serious.

Few women exposed misinformation conveyed by their HCPs who may lack current HPV information. A woman reported a provider has recommended her cesarean section because of her vaginal warts.

#### Taking multidisciplinary approach

More than half of the women interviewed had seen at least three doctors in the 12 months before the interview. It seems that the more women went to different clinics (offices), the more HPV-knowledge and less stress. However, this would have cost more. Women reported attending and talking to a number of different HCPs. According to participants’ point of view, there is not an optimal cross-disciplinary referral system among gynecologists and other HCPs. A woman with oral ulcers who was worried that her lesion might be an HPV-related oral cancer (or precancerous) stated: *“I’ve seen an ENT specialist and a dentist to get other opinions just to be on the safe side.”*(W.28).

Another woman expressed:

*"When my partner and I realized we have genital warts, we did not know which specialist to go to. We went to an infectious disease specialist, urologist, gynecologist and dermatologist. In my opinion, the best specialist in the treatment of genital warts is a dermatologist. I wish we had gone to a dermatologist from the very beginning of my diagnosis."* (W.14)

## Discussion

In this study, experiences, needs and preferences of HPV-infected women about patient-providers communication clearly identified. Findings indicated that providers’ communication strategies are as important as content. The way that HPV-information is conveyed needs careful consideration. Similar findings showed how providers communicate women about their HPV results can have an influence on their emotional responses to HPV diagnosis [34]. Women may also be less able to accurately weigh the benefits and harms of CC screening due to a lack of understanding [8]. In agreement with our finding, a study implied using clear, evidence-based communication in delivering HPV results and information will help women understand their health condition [7].

Our findings noted that healthcare providers should be cautious in using cancer-related terms. Similarly, ASCCP consensus (2020) is hesitant that precancer is the best definition for CIN2 or CIN3 and higher [35, 36]. Using new risk tables, to risk estimates, and management of abnormal screening tests, which are freely available online at https://CervixCa.nlm.nih.gov/RiskTables, [37] could alter providers and patients’ interpretation of positive HPV results’ risk to the factual extent.

In response to participant’s need HCPs to spend enough time answering their questions, our findings suggested addressing current gaps and uncertainties in HPV-knowledge. Similarly, another study addressed the importance of disclosing existing limitations in HPV-knowledge with patients [38]. Another study indicated HCPs may be faced with HPV- related questions from patients [26]. Due to the limited number of gynecologist-oncologists, more time should be allocated for patient education and counseling in the primary level of care. Previous studies suggested that to provide basic HPV-information, waiting room posters or leaflets or an educational website could be beneficial. Therefore, more time would leave to face-to-face appointments to answer questions and make a patient-centered two-way HPV communication [22, 39].

In line with our findings that women needed to be treated gently and respectfully in clinical appointments and gynecological examination, a qualitative study conducted in Tanzania (2019), indicated that the gynecological examination was an element that may hinder women from returning to screening [40]. In the screening setting, HPV testing may lead to questions regarding sexual history, which clinicians should be prepared to discuss [41]. Iranian religious beliefs that physicians may hold about sexuality can be a barrier to optimal sexual health care [42]. In our setting, midwives are qualified communicators because they are of the same sex and are trained for sexual counseling.

One of our findings that may be influenced by Iran’s religious context was that women viewed the protective behavior of HCPs as the rejection related to impurity (being dirty) issues. Another study indicated that HPV patients may perceive certain physician behavior as negative even though the doctor does not plan to [41]. In a qualitative study, emphasizing cancer screening to take the focus away from HPV’s association with sexuality has been suggested [43]. HCPs in societies with religious-cultural sensitivity may face more challenges in informing patients and moderating patients’ psychosocial response. A study emphasized that presenting information should be in ways that are sensitive to a range of cultural norms and social values associated with sexual activity to achieve better health outcomes [44]. Particularly in culturally sensitive societies, providers should also be aware of the privacy and secrecy issues associated with an STD diagnosis, because privacy is a key factor of high-quality primary services [16]. Another issue that HCPs should consider is the ethics of biological research and patient consent to participate in HPV clinical trials, which our results emphasize.

In a study conducted in Kenya, financial support was key facilitators of HPV-positive women’s treatment access [45]. Due to the high cost of colposcopy in Iran, referral of women to government-funded clinics (by HCPs) may improve women’s adherence to treatment. Iranian health insurance providers are suggested to include CC screening procedures in their service packages [46].

Our study showed that knowledgeable and competent HCP is among the main needs of women infected with HPV. Provision of communication skills training for sample takers should help minimize adverse psychological responses [7]. Counseling strategies that incorporate many dimensions of communication will probably be helpful to primary care providers [44]. Yet most physicians receive limited training in communication skills. Policy makers and stakeholders can leverage training grants, payment incentives, certification requirements, and other mechanisms to develop and reward effective patient-centered communication [47]. As previous studies have emphasized, doctors, medical students, and dental providers need training to provide appropriate basic HPV-knowledge to patients [19, 28, 48, 49]. HCPs also should note that HPV may contaminate protective equipment but HPV-DNA transfer to medical personnel is unlikely to occur [50]. Gynecologists’ awareness of the provision of the HPV-DNA test, such as time intervals and test series, was unsatisfactory in another study. It was because of the multiplicity and rapidly evolving guidelines [51]. Potential overuse of HPV testing among women’s health providers was reported in another study [52]. This may indicate the patient-centered care or knowledge deficiency. Further studies are needed to characterize compliance of providers with national guidelines for HPV testing.

Service coordination among different levels of care was addressed in another study [27]. In our study, since the most women interviewed had visited at least three doctors, Iranian physicians should follow a multidisciplinary approach and, knowing their own limitations, refer patients to proper specialist facilities as a first step towards effective management.

We acknowledge the study’s limitations. Our study environment was one busy specialized center for HPV-patients so it is possible that emerged themes would be different from other clinics nationally. Therefore, caution is required when interpreting data. We should comment on the potential influence of a highly educated sample (~ 60% with university education) as another limitation. Naturally, qualitative research is not targeted at representativeness, but it seems likely that less-educated women’s viewpoints were not thoroughly explored. Some of the findings are cultural-specific and cannot be generalized to all communities.

Like other qualitative studies, the generalizability and relative weight of emergent categories are not obvious. This paper follows COREQ checklist for reporting qualitative studies [53]. Participants in this study may have been more comfortable talking about HPV. They may also have personal prejudices towards doctors or other care providers, which may affect their experiences and expectations. Findings do not necessarily generalize health care in developing countries.

There is a need for further development of research on patient-provider communication, in particular with respect to a more solid theoretical basis, integration of methods including qualitative and quantitative methods, self-evaluations, and interaction analyses, and also concerning conducting more longitudinal studies.

## Conclusions

Our findings, based on the experiences and perceptions of HPV women, contain messages that may provide deeper insight to HCPs working with HPV-infected people to establish evidence-based strategies to support and facilitate patient-provider communication in the primary and specialized levels of care. These practical tips will improve the quality of care for HPV-positive women, which is an integral component of cervical cancer prevention programs.

- Women need advice on boosting immunity, vaccinations, follow-up, and sexual health.
- HPV-positive women’s main expectations of their healthcare providers include communication and counseling skills, commitment to professional principles, and HPV-knowledge.
- Exposing current HPV-knowledge limitations, gaps and uncertainties prevent women from excessive clinical referrals.

## Supplementary Information

**Additional file 1.**

## Data Availability

The data that support the findings of this study are available from the corresponding author, [STM], upon reasonable request.

## Abbreviations

HPV: Human Papillomavirus
CC: Cervical cancer
HCPs: Health Care Providers
