# Supplementary material for: Understanding HPV-positive women’s needs and experiences in relation to patient-provider communication issues: a qualitative study
Source: BMC Health Serv Res. 2021 Mar 31;21:286. doi: 10.1186/s12913-021-06283-w (PMC8011207; doi:10.1186/s12913-021-06283-w)
Supplement: Supplementary file 1 — Additional file 1. [file 12913_2021_6283_MOESM1_ESM.docx]

Title: Understanding HPV-positive women's needs and experiences in relation to patient-provider communication issues: A qualitative study

Kowsar Qaderi^1^, Mehrnaz Geranmayeh^2^, Farnaz Farnam^2^, Shahrzad Sheikh Hasani^3^, Seyedeh Tahereh Mirmolaei^2*^

**Additional file 1**

Semi-structured interview guide:

An open question about the management of information and advice given by HCPs:

“What did your healthcare provider tell you about HPV at the time of diagnosis?”

An open question about experiences of being told the diagnosis by HCPs:

“Please tell me about your experiences, thoughts, and feelings during the communication with your clinician or doctors?” and “What makes you feel better or worse when you are thinking about patient-provider communication in different setting (Lab, clinic or hospital)?”

We encouraged patients to express their preferences and considerations to improve the provider/patient communication asking:

“How do you prefer caregivers to treat you or talk to you about your condition??” and “What are your preferences and expectations related to healthcare providers?”

To sought healthcare providers' perspectives on needs and preferences of HPV-positive women about the provider/patient communication, we asked them: “In your experience, women with HPV prefer how healthcare providers treat them?”

Open questions such as "What do you mean?" and "Please expand" were used to extract more clear and detailed responses.
